# Editorial: Global excellence in fungal pathogenesis: Central and South America

**DOI:** 10.3389/fcimb.2024.1481806

**Published:** 2024-09-11

**Authors:** Lysangela R. Alves, Clayton Luiz Borges, Fausto Almeida

**Affiliations:** ^1^ Gene Expression Regulation Laboratory, Instituto Carlos Chagas - Fiocruz, Curitiba, Brazil; ^2^ Laboratory of Molecular Biology, Institute of Biological Sciences, Federal University of Goiás, Goiânia, Brazil; ^3^ Department of Biochemistry and Immunology, Ribeirao Preto Medical School, University of Sao Paulo, Sao Paulo, Brazil

**Keywords:** fungal infection, South America, Central America, sporothichosis, *Cryptoccocus neoformans*, *Cryptococcus gattii*, paracoccidioidomycosis, extracellular vesicles (EVs)

Central and South America are regions with a high prevalence of significant fungal diseases such as paracoccidioidomycosis (PCM), coccidioidomycosis, sporotrichosis, histoplasmosis, cryptococcosis, and others (Laniado-Laborín et al., 2019; Lockhart and Guarner, 2019; Araúz and Papineni, 2021; Hahn et al., 2022; Zhao et al., 2023). These diseases impose a substantial burden on local healthcare systems and have a significant impact on the quality of life for affected populations. Furthermore, the region’s diverse ecological and climatic conditions can influence the distribution, virulence, and epidemiology of pathogenic fungi, emphasizing the necessity for region-specific studies. Understanding the interaction between these environmental factors and fungal pathogens is crucial for developing improved strategies for the prevention, diagnosis, and treatment of fungal diseases in Central and South America (Almeida et al., 2019; Lockhart and Guarner, 2019; Rodrigues and Nosanchuk, 2020).

## Sporotrichosis

Sporotrichosis, caused by fungi of the *Sporothrix schenckii* complex, represents a significant public health concern in Central and South America. This subcutaneous mycosis primarily affects individuals through traumatic inoculation of fungal spores from environmental sources such as soil, plants, and organic matter. In recent years, zoonotic transmission, particularly from infected cats which emerged as a significant route of infection (da Rosa et al., 2005; Barros et al., 2011; Rodrigues et al., 2016).

Brazil is the most affected country by sporotrichosis, with a recent dramatic increase in cases, resulting in thousands of reported cases annually. This surge is largely attributed to the zoonotic transmission from domestic cats, which can develop severe forms of the disease and transmit it to humans through scratches or bites. A study conducted in Rio de Janeiro documented over 4,000 human cases, highlighting the endemic nature of sporotrichosis in this area (Schubach et al., 2002; Barros et al., 2011; Macêdo-Sales et al., 2018). In Central and South America, public health measures have concentrated on controlling the zoonotic transmission of sporotrichosis, with a particular emphasis on the role of cats as a source of infection. Veterinary health initiatives include the treatment and management of infected animals and public awareness campaigns designed to educate communities about transmission risks and the importance of seeking medical and veterinary care. The education of pet owners and the promotion of animal health have significantly impacted the management of infected animals. These efforts are crucial in reducing the incidence of human sporotrichosis and controlling outbreaks (Schubach et al., 2004; Kauffman et al., 2007; De Souza et al., 2024).

## Cryptococcosis

Cryptococcal infections, primarily caused by *Cryptococcus neoformans* and *Cryptococcus gattii*, represent significant opportunistic fungal infections in Central and South America (Escandón et al., 2018; Firacative et al., 2018; Lizarazo and Castañeda, 2022). These infections predominantly affect immunocompromised individuals, particularly those with HIV/AIDS, but can also occur in immunocompetent hosts. Cryptococcal meningitis, the most severe manifestation, is a leading cause of morbidity and mortality among HIV-infected individuals in the northeastern region of Brazil (Oliveira et al., 2023).

In Brazil, cryptococcal infections are a major health concern, with estimated incidence rates of approximately 3 to 5 cases per 100,000 inhabitants annually. This incidence is markedly elevated in populations with a high prevalence of HIV. A study conducted in Brazil highlighted that cryptococcal meningitis accounts for approximately 6-10% of all AIDS-related deaths, emphasizing the significant impact of this infection on the healthcare system (Dos Reis et al., 2022; Oliveira et al., 2023).

*Cryptococcus gattii*, which primarily affects immunocompetent hosts, has also been increasingly reported in Central and South America (Lizarazo and Castañeda, 2022). This species is associated with environmental exposure to eucalyptus trees and soil. In Colombia, the incidence of *C. gattii* infections has risen, particularly in regions with favorable environmental conditions for the fungus. The clinical presentation of *C. gattii* infections can be more severe than *C. neoformans*, often leading to pulmonary infections and disseminated disease (Escandón et al., 2018; Firacative et al., 2018).

## Paracoccidioidomycosis

Paracoccidioidomycosis (PCM) is a systemic fungal infection endemic to Central and South America, caused by the dimorphic fungi *Paracoccidioides* spp., which consists of five species: *P. brasiliensis*, *P. americana*, *P. restrepiensis*, *P. venezuelensis*, and *P. lutzii* (Marques, 2013; Martinez, 2017; Turissini et al., 2017). This disease predominantly affects rural workers who are exposed to the fungal spores present in the soil. PCM poses a significant public health challenge in the region, given its chronic nature and potential for severe morbidity if not properly diagnosed and treated (Peçanha et al., 2017; Shikanai-Yasuda et al., 2017).

In Brazil, PCM is highly prevalent, with incidence rates ranging from 1 to 3 cases per 100,000 inhabitants annually, accounting for over 80% of reported cases in Latin America (Mendes et al., 2017). The disease is particularly prevalent in the southeastern, southern, and central-western regions of the country, where environmental conditions favor the growth of *Paracoccidioides* spp (Peçanha et al., 2017). Infection typically occurs via the inhalation of conidia, which transits into the pathogenic yeast form in the host’s lungs. Subsequently, the infection may disseminate to other parts of the body, resulting in the formation of granulomatous lesions in organs such as the lungs, skin, mucous membranes, and lymph nodes (Marques, 2013; Shikanai-Yasuda et al., 2017). The impact of PCM in Central and South America extends beyond individual morbidity, affecting healthcare systems and economies. Rural communities, where the disease is most prevalent, often have limited access to healthcare facilities and diagnostic tools, leading to delays in diagnosis and treatment. Public health initiatives aimed at increasing awareness, improving diagnostic capabilities, and ensuring the availability of effective antifungal medications are crucial for the management and reduction of the impact of PCM in these regions (Shikanai-Yasuda et al., 2017; Hahn et al., 2022).

The objective of this editorial is to advance global knowledge of fungal pathogenesis, offering valuable insights applicable to other regions facing similar challenges. The researchers contributing to this Research Topic presented four themed articles highlighting the latest advances in our understanding of fungal pathogenesis in PCM and sporotrichosis.

Professor Loures coordinated two studies aimed at understanding the immune regulatory mechanisms involved in Paracoccidioidomycosis (PCM), a systemic fungal disease endemic in Latin America caused by *Paracoccidioides brasiliensis*. In the study by Borges et al., the researchers delve into the intricate dynamics between the immune system and *P. brasiliensis* within granulomatous lesions, crucial in the defense against PCM. The objective of the research was to identify essential genes and proteins that are activated under stress conditions through the analysis of transcriptional and proteomic data from yeasts within pulmonary granulomas in a mouse model. Key findings revealed a compromised lung environment, characterized by altered gene expressions related to muscle function and an intensified immune response involving various immune cells and inflammatory mediators. It is noteworthy that the study identified novel immune regulatory mechanisms within granulomatous lesions, suggesting complex interactions between the fungus and host immune proteins including CTLA-4, PD-1, and arginase-1. In addition, the discovery of iron’s critical role in granuloma function, facilitated by fungal siderophores, provides novel insights into fungal virulence mechanisms in this specific context. Overall, this research advances our comprehension of PCM pathogenesis by elucidating the roles of immune regulation and iron metabolism within granulomatous structures. Meanwhile, in the second study coordinated by Professor Loures, Kaminski et al. explore how specific pathogen recognition receptors (PRRs), namely Dectin-1, TLR2, and TLR4, influence the suppressive activity of myeloid-derived suppressor cells (MDSCs) in PCM. Key findings indicate that these PRRs play crucial roles in inducing the expression of immunosuppressive molecules such as PD-L1, IL-10, and nitrotyrosine, by MDSCs upon fungal challenge. The study employed *in vitro* and *in vivo* approaches, including antibody treatments and genetically modified mice lacking these receptors, to demonstrate their impact on MDSC function and subsequent suppression of T cell proliferation. By uncovering this intricate network of PRR signaling and its role in immunosuppression, the research provides valuable insights into the mechanisms that regulate host immunity and disease severity in pulmonary PCM. Collectively, these two articles may advance the development of targeted therapies aimed at modulating these pathways to enhance immune responses against PCM and potentially other fungal infections with analogous immune evasion strategies.

In this review, Morgado et al., identify significant gaps in the global understanding and preservation of *Sporothrix* species, which cause sporotrichosis, a subcutaneous mycosis. The study underscores the importance of polyphasic taxonomy and molecular identification for accurate species classification and epidemiological studies. The most significant findings include the prevalence of *S. globosa*, *S. schenckii*, and *S. brasiliensis* as the most identified species associated with sporotrichosis. However, the review reveals a critical issue: only 17% of *Sporothrix* isolates worldwide are preserved in culture collections, indicating potential challenges in accessing and utilizing these strains for future research. This underscores the necessity for improved efforts in both documenting and preserving diverse *Sporothrix* strains to support ongoing and future studies on this medically important fungal group.

The study by Dallastella et al., which includes one of us, provides a comprehensive overview of RNA-binding proteins (RBPs) and their roles within fungal extracellular vesicles (EVs). Traditionally recognized for their intracellular functions, RBPs have been found in EVs from various mammalian cells, indicating a broader role. In fungi, EVs contain a variety of proteins involved in RNA synthesis, folding, degradation, and translation regulation. These include RNA helicases, aminoacyl-tRNA synthetases, and canonical RNA-binding domains like RRM, Zinc finger, and KH domains. These proteins are essential for RNA processing and transport, indicating the existence of a conserved mechanism for packaging RNA cargo into fungal EVs. The presence of polyadenylate-binding protein (PABP) in fungal EVs underscores its role in mRNA regulation, influencing processes such as translation, stability, and localization. Moreover, the authors recognize the current gaps in knowledge regarding the specific processes that determine which RNA molecules are selected for release into EVs. This review synthesizes current knowledge of RBPs and RNA metabolism in fungal EVs, suggesting avenues for future research to explore the molecular mechanisms governing RNA sorting and function in these vesicles across different fungal species.

In conclusion, this Research Topic advances our understanding of the immune response against PCM; identifies challenges in determining the frequency of Sporothrix species in culture collections; identifies gaps in molecular identification; and reviews the characterization of RBPs and RNA metabolism-related proteins in fungal EVs.
